# Toward COVID-19 Information: Infodemic or Fear of Missing Out?

**DOI:** 10.3390/healthcare8040550

**Published:** 2020-12-10

**Authors:** Sen-Chi Yu, Hong-Ren Chen, An-Chia Liu, Hsin-Yu Lee

**Affiliations:** 1 Department of Counseling and Applied Psychology, National Taichung University of Education, Taichung City 40306, Taiwan; angie811381537@gmail.com (A.-C.L.); julia88114@gmail.com (H.-Y.L.); 2 Department of Digital Content and Technology, National Taichung University of Education, Taichung City 40306, Taiwan

**Keywords:** coronavirus disease 2019 (COVID-19), infodemic, fear of missing out, social media, social network sites

## Abstract

Coronavirus disease 2019 (COVID-19) has caused a global pandemic and exerted a profound physiological and mental impact on the public. Due to anxiety from being bombarded by information from the news and social media, people may constantly read and repost, with a fear of missing out (FOMO), information about COVID-19 on social media. So far, there has been little research on COVID-19 FOMO. We therefore compiled the COVID-19 information fear of missing out scale (CIFS) and administered it to 1178 adults in Taiwan to identify the possible factors influencing CIFS scores. We demonstrated that the CIFS had good reliability, factor validity, and criterion validity. With regard to demographic variables, we found that gender, marital status, travel time to the nearest hospital, and educational background influenced CIFS scores. In contrast, the participant age and whether he or she lived in an urban area did not affect the CIFS scores. With regard to social media usage, social media usage time (*r* = 0.025) and the numbers of COVID-19-related posts read on social media (*r* = 0.117) or instant messaging (*r* = 0.169) were not highly correlated with CIFS scores. Rather, CIFS scores were found to be significantly correlated to the frequency of reposting COVID-19-related information on social media (*r* = 0.497) and on instant messaging (*r* = 0.447). These results indicate that CIFS scores are closely associated not with passive browsing on social media but with the frequency at which an individual actively reposts information. In other words, what creates CIF is not an overabundance of information (i.e., an infodemic) but the active reposting and interpretation of information. Individual autonomy for interpretation of the received information and self-determination about reposting are key factors for COVID-19 information FOMO. When facing the COVID-19-related news on social media, it is the active information-related FOMO, not the passive infodemic, that influences our social media usage.

## 1. Introduction

Coronavirus disease 2019 (COVID-19) is an infectious disease caused by a coronavirus. It was first identified in December 2019 in Wuhan, China, and has since become a severe global threat [1]. As of 7 July 2020, more than 11.6 million infections were confirmed around the world, resulting in at least 538,000 deaths [2].

Not only has COVID-19 had a physiological impact on the general public, it has also caused psychological anxiety. Due to social media, the impact and speed of information dissemination regarding COVID-19 are vastly different from those during pandemics in previous centuries. The WHO even coined a new term, “infodemic”, defined as “an overabundance of information—some accurate and some not—that makes it hard for people to find trustworthy sources and reliable guidance when they need it” [3]. An infodemic refers to excessive information spreading as rapidly as an epidemic and causing anxiety and panic. Thus, the mental health issues created by an infodemic are worth investigating further.

Some would argue that our anxiety is fueled in part by the wealth of information that social media provides [3]. The convenience of social media enables people to continuously search for relevant news and repost information on social media or instant messaging mobile apps in hopes of getting a grasp on information on COVID-19 and reducing their own risk of infection. However, instead, the constant use of social media is causing a fear of missing out (FOMO). This study proposed a novel latent construct: COVID-19 information FOMO (CIF), which is the anxiety that an individual feels about control over information as COVID-19 spreads. This FOMO. with regard to important information, is similar to conventional socially related FOMO: a worried feeling that you may miss some events that other people are going to, especially caused by things you see on social media [4].

CIF and socially related FOMO both involve FOMO on important information and opportunities. However, COVID-19 information-related FOMO specifically involves disease-related information and preventive health opportunities, whereas socially related FOMO involves social information and opportunities. Socially related FOMO is defined as a pervasive anxiety that others might be having rewarding experiences from which one is absent; socially related FOMO is characterized by the desire to stay continually connected with what others are doing [4]. This study defined CIF as “a pervasive anxiety caused by the FOMO on any disease-related information and the display of a continuing need to maintain control over disease-related information”.

Some studies have indicated that socially related FOMO is associated with social media use, depression, and anxiety [4,5,6]. The aim of this study was to develop and validate the COVID-19 information FOMO scale (CIFS) and investigate how CIF is related to social media use, depression, anxiety, and demographic variables.

## 2. Materials and Methods

### 2.1. Participants and Procedures

The survey targeted Taiwanese adults aged over 20. We recruited a total of 1178 samples in Taiwan obtained by convenient sampling (ages ranging from 20–78, mean age = 29.79, standard deviation = 10.76, females = 70.4%, males = 29.6%). A digital link to our questionnaire (via Google Forms) was post on online forums and Facebook. The introduction to the questionnaire explained our policy on privacy protection for all participants. Participants who completed the questionnaire could enter a prize draw, and winners were awarded electronic gift certificates. No conflicts of interest were present in the current study. The Institutional Review Board of National Chung Cheng University granted ethical approval to carry out the study (Ethical Application Ref: CCUREC-109051101).

### 2.2. Measures

#### 2.2.1. COVID-19 Information FOMO Scale (CIFS)

##### Development of CIFS

The definition of socially related FOMO emphasizes anxiety from FOMO on social relationships and potential benefits. This study focuses on the anxiety that individuals have regarding their control over information on COVID-19 on the internet. We therefore defined CIF as “a pervasive anxiety caused by the FOMO on any disease-related information and the display of a continuing need to maintain control over disease-related information”.

Socially related FOMO cites the self-determination theory (SDT) [7] to explain the cause of FOMO. According to the SDT, effective self-regulation and psychological health are based on the satisfaction of three basic psychological needs: competence, autonomy, and connectedness with others. When said needs of an individual are not met, he or she may turn to social media because social media is a resource for getting in touch with others, a tool to develop social competence, and an opportunity to deepen social ties. We similarly used the SDT to explain CIF, in which individuals used social media to obtain the latest epidemiological information and maintain their own competence and autonomy. They also use social media to keep in touch with others, which includes reposting the latest information provided by other people.

Based on relevant literature on FOMO and the psychological influences of COVID-19, we complied our 6-item, 4-point CIFS. Table 1 displays the scale items and their factor loadings.

##### Psychometric Properties of the CIFS

We used two-phase factor analysis to factor the validity of the CIFS. We randomly split the data into Group 1 (*n* = 579) and Group 2 (*n* = 579). We conducted exploratory factor analysis (EFA) on Group 1 and confirmatory factor analysis (CFA) within the framework of structural equation modeling on Groups 2. EFA serves to identify the factor, and the factor was later confirmed using CFA with the LISREL 8.80 software program (Available from Scientific Software, Lincolnwood, IL, USA). A CFA was run to test whether the six items identified in previous EFA exhibited a single construct.

We applied EFA using principal axis extraction. A value of 0.35 was determined as a viable cut-point for judging factor loadings [8]. The CIFS exhibited one factor structure. Factor loadings of items are shown in Table 1.

**Figure 1:**
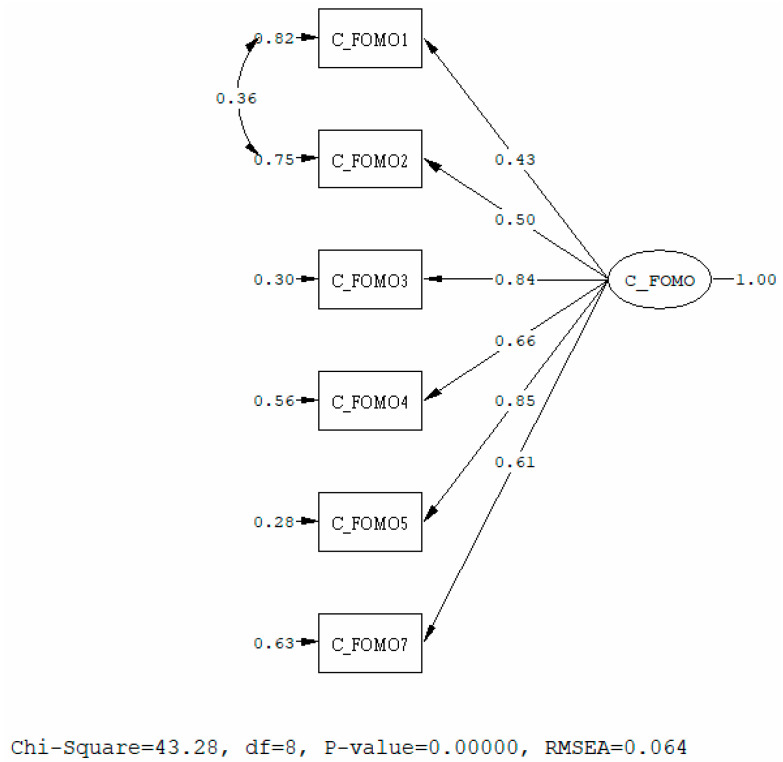
Confirmatory factor analysis on CIFS.

A CFA was then run to verify the structure of the CIFS. The original model showed a mediocre fit (Chi-Square = 281.98, df = 9, *p*-value = 0.00, root mean square error of approximation (RMSEA) = 0.167, 90% CI for RMSEA = (0.15; 0.18), comparative fit index (CFI) = 0.92, standardized root mean square residual (SRMR) = 0.076). The modification index (MI) provided by statistical software indicates that error covariance should be added between item 1 and 2. Correlated errors indicate that items are highly correlated and that unique variances of the associated indicators overlap. Correlated errors are commonly used in model re-specification strategies of theoretical rationale [9]. Since the meaning of items 1 and 2 are related in meaning, we added correlated errors between the two items. The revised model showed good fit (Chi-Square = 43.28, df = 8, *p*-value = 0.00, RMSEA = 0.064, 90% CI for RMSEA = (0.017; 0.055), CFI = 0.99, SRMR = 0.019). The Cronbach’s alpha of the CIFS was 0.818, indicating good reliability. The results showing the standardized solution of SEM coefficients are presented in Figure 1.

##### Criterion-Related Validity of CIFS

FOMO is a type of situational anxiety that should be associated with mental health variables, such as general anxiety and depression [4], and more correlated with general anxiety than with depression. For instance, the coefficients of the correlation between FOMO and depression and between FOMO and anxiety were 0.40 and 0.49, respectively [5]. The coefficients of the correlation between fear of COVID-19 and depression and between fear of COVID-19 and anxiety were 0.425 and 0.511 [6]. In this study, the coefficients of the correlation between the CIFS and the 10-item Center for Epidemiologic Studies depression scale (CES-D) and between the CIFS and the 7-item general anxiety disorder scale (GAD-7) were 0.293 and 0.403. These results support the criterion-related validity of the CIFS.

#### 2.2.2. The 7-Item General Anxiety Disorder Scale (GAD-7)

The GAD-7 is a seven-item and four-point scale measuring the symptoms of anxiety over 2 weeks [10]. We translated the GAD-7 into Mandarin Chinese. The GAD-7 had good reliability, as well as criterion, construct, and factorial validity [10]. The Cronbach’s alpha of our GAD-7 was 0.933, indicating good reliability.

#### 2.2.3. The 10-Item Center for Epidemiologic Studies Depression Scale (CES-D)

The CES-D is one of the most widely used depression scales in the world [11] and is verified for reliability and validity in a number of languages [12]. The original CES-D is a four-point, 20-item scale. In addition to the original full 20-item CES-D, various investigations have proposed shorter forms. This study used a 10-item Boston form (CESD-10) with a four-point Likert type originally developed by Kohout et al. [13] and translated into Chinese by Yu et al. [9]. For this study, the CES-D had a coefficient alpha of 0.952, indicating good reliability.

## 3. Results and Discussion

### 3.1. The CIFS Related to Demographics and Individual Differences

We investigated whether CIFS scores varied with age, gender, education background, marital status, place of residence, and travel time to the nearest hospital by car. A summary of ANOVA results is shown in Table 2.

The correlation between the CIFS score and age was 0.126 (*p* < 0.05), indicating a very weak correlation. Women (M = 9.68, SD = 2.93) had significantly lower CIFS scores than men (M = 10.16, SD = 3.86, F (1, 1273) = 6.016, *p* = 0.014 < 0.05). This result was similar to that obtained by Qutishat and Sharour [14], which explored social media FOMO among Oman university students and found that males scored a higher level of fear of missing out than females. However, this result was inconsistent with that derived by Lee regarding coronavirus anxiety [15], which indicated that coronavirus anxiety was negatively correlated with age and positively correlated with educational background but not correlated with gender. The inconsistency between these results may be due to the fact that this study focuses on anxiety associated with searching for information on COVID-19, whereas Lee’s study focused on anxiety about COVID-19 itself. The different anxiety sources led to different results.

The CIFS scores varied by the highest level of education (F (2, 1272) = 7.019, *p* = 0.001 < 0.05). The Scheffe post hoc procedure indicated that those who have a bachelor’s degree (M = 9.69, SD = 3.14) and those who have a postgraduate degree (M = 9.92, SD = 3.31) score lower than those who had a senior high school diploma or lower (M = 11.27, SD = 4.22). There was no significant difference in scores of the CIFS between those with bachelor’s degrees and those with postgraduate degrees. Our results revealed that more educated individuals were more able to discern the accuracy of information sources and possessed more healthcare knowledge, which led to less anxiety.

The CIFS scores varied by marital status (F (2, 1272) = 13.327, *p* = 0.000 < 0.05). Married individuals (M = 10.70, SD = 3.78) scored significantly higher than single individuals (M = 9.59, SD = 3.07), but the scores of the divorced individuals (M = 9.37, SD = 2.19) showed no significant differences from those of the single or married individuals, which may be due to the small sample size (*N* = 19). There was originally a “widowed” option, but no participants chose this option, so it was not included in our analysis. We infer that married individuals may feel greater anxiety than single individuals about searching for information because they already have a spouse or children and fear that their family members may contract COVID-19.

The CIFS scores did not vary by place of residence, (F (2, 1269) = 1.906, *p* = 0.149 > 0.05). That is, whether the participant lived in an urban, rural, or remote area made no significant difference. However, the CIFS scores did vary with travel time to the nearest hospital by car, (F (2, 1271) = 11.057, *p* = 0.000 < 0.05). Significant differences were found among short distances (less than 30 min to the nearest hospital) (M = 9.72, SD = 3.11), moderate distances (31–59 min to the nearest hospital) (M = 10.46, SD = 3.77), and long distances (more than 1 h to the nearest hospital) (M = 12.89, SD = 6.03). This study found that an individual living in an urban area did not affect his or her CIFS score. Rather, what really influenced the CIFS score was how far the nearest hospital was. This may involve how long it would take to get to the hospital in the event of an emergency and may be associated with the residential patterns and health insurance system in Taiwan. Taiwan is small and densely populated, and in even in some non-urban areas, it rarely takes more than 1 h to get to the nearest large-scale hospital. Thus, what truly affects CIFS scores is how long it takes to get to the nearest hospital rather than whether one’s place of residence is in an urban area. Enrollment in the state-run health insurance system is mandatory in Taiwan. At present, medical expenses incurred by COVID-19 are covered by the government. As a result, what influenced CIFS scores was not whether an individual had health insurance but how far he or she lived from the nearest hospital.

### 3.2. The CIFS and Social Media Use

The correlation between CIFS scores and Facebook usage time and Instagram (IG) usage time were 0.025 (*p* > 0.05) and 0.003 (*p* > 0.05), respectively, which did not reach the level of significance. Thus, CIFS scores were not associated with the overall usage time of social media. A summary of correlation for CIFS Scores and social media use is shown in Table 3.

The correlation between CIFS scores and “number of COVID-19-related posts read daily on Facebook”, “number of COVID-19-related posts read daily on IG”, and “number of COVID-19-related posts read daily on instant messaging (such as LINE, WeChat, and Telegram)” were 0.117 (*p* < 0.05), 0.169 (*p* < 0.05), and 0.140 (*p* < 0.05), respectively. Despite their significance, they only presented low degrees of correlation. In contrast, the correlation between CIFS scores and “frequency of reposting COVID-19-related information on social media” and between CIFS scores and “frequency of reposting COVID-19-related information on instant messaging” were 0.497 (*p* < 0.05) and 0.447 (*p* < 0.05), both reaching the level of significance. These results show that CIFS scores were closely associated, not with passive browsing on social media or instant messaging, but with the frequency at which an individual actively reposted or shared information. In other words, what creates CIFS scores is not an overabundance of information (an infodemic) but the active reposting and interpretation of information.

This result can be explained using the SDT [7], which holds that individual behavior is subject to the influence of autonomy, competence, and relatedness. Compared to passive information browsing, active interpretation and reporting information indicate greater autonomy. When individuals have greater self-determination with regard to their own activities, they have greater control over their own behavior. Reposting behavior also displays competence, which is the feeling of having control over both internal and external environments. Compared to passive browsing, reposting important information for other people is a display of greater competence. The targets of reposts are generally one’s own friends and family, which promotes relatedness. By sharing information, they strengthen their ties with other people.

This result is inconsistent with that derived by Cinelli et al. [16], which presented that providing excessive information on social media amplifies the infodemic. The dissemination of information is determined by the characteristics of the social media themselves, and the effects of the infodemic vary with the media. The results of this study found that it is the usage behavior (passively receiving information and actively reposting information) that makes the difference, not the social media.

## 4. Limitation

This study is subject to the following limitations. With regard to participant gender, there were more women than men in this study. This may be because the questionnaire was more health-oriented and women are more willing to respond to such questions. In terms of time, this study was conducted in July 2020. By this time, Taiwan had already maintained a pretty long stretch of zero new confirmed cases and was past the peak of the epidemic. Compared to many other countries around the world, where the numbers of new confirmed cases remain high, the general public in Taiwan is already less anxious about COVID-19 than they were during the peak of the epidemic. Longitudinal follow-up research should be conducted to understand the influence of time on CIFS scores.

With regard to sample collection, the samples of this study were collected in Taiwan. Geographically speaking, Taiwan is very close to China, and they have close business and tourism relations. Originally, Taiwan was at a very high risk of an outbreak, but the government’s precautionary measures early on allowed them to properly handle and control the epidemic. With regard to information dissemination, the government passed laws to penalize the spread of false information, and this may have reduced the dissemination of unconfirmed rumors. Furthermore, the government held daily press conferences about the epidemic to notify the public about new confirmed cases and other information, and the daily announcements of official information may have reduced the anxiety of the public. In Taiwan, the government covers the expenses of the National Health Insurance. To prevent cover-ups, the government also bears all of the expenses for COVID-19 treatment. All of these factors above may have influenced the CIFS scores. Thus, transnational comparisons and longitudinal studies are suggested for future research to gain a more complete understanding of the applicability of the CIFS.

## 5. Conclusions

This study developed and validated the COVID-19 information fear of missing out scale (CIFS) and administered it to 1178 adults in Taiwan to identify the possible factors influencing CIFS scores. The analytical results showed that the CIFS has good reliability, factor validity, and criterion validity.

We found that being men, being married, long travel time to the nearest hospital, and high educational background were risk factors for COVID-19 information FOMO. On the contrary, age and living in an urban area or not did not affect CIFS scores.

With regard to social media usage, CIFS scores significantly correlated with the frequency of reposting COVID-19-related information on social media. However, the numbers of COVID-19-related posts read and social media usage time had weak correlation with CIFS scores. Rather, these results indicate that CIFS scores are associated with the frequency of active reposts rather than passive browsing. In other words, it is the active reposting of, not passive receiving of, the overabundance of information (i.e., infodemic) that reflects COVID-19 information FOMO. Individual autonomy for interpretation of the received information and self-determination about reposting are key factors for COVID-19 information FOMO.

## Figures and Tables

**Table 1:** Factor loadings of the COVID-19 Information Fear of missing out scale (CIFS).

	Item	Factor Loading of EFA	Factor Loading of CFA
1	I start searching for information on COVID-19 whenever I use my phone.	0.54	0.35
2	I am afraid of missing out on the latest information on COVID-19.	0.60	0.45
3	I feel anxious if my friends do not share information on COVID-19 with me.	0.79	0.53
4	I think I spend too much time on following the latest information on COVID-19.	0.66	0.50
5	I feel anxious if I do not read all of the posts associated with COVID-19 on social media.	0.80	0.51
6	I share health information on preventing COVID-19 on social media.	0.62	0.47
Cronbach Alpha		0.818	

**Table 2:** Summary of ANOVA results on CIFS scores.

Factor	Level	M	SD	F	*p*-Value	Post-Hoc Tests
Gender	1. Male	9.68	2.93	6.016	0.014 *	2 > 1
	2. Female	10.16	3.86			
Education Level	1. Senior high	11.27	4.22	7.019	0.001 **	1 > 2
	2. Bachelor	9.69	3.14			1 > 3
	3. Postgraduate	9.92	3.31			
Marital Status	1. Married	10.70	3.78	13.327.	0.000 ***	1 > 2
	2. Single	9.59	3.07			
	3. Divorced	9.37	2.19			
Place of Residence	1. Urban	9.79	3.19	1.906	149	
	2. rural	9.96	3.43			
	3. Remote area	11.70	5.87			
Travel Time to Nearest Hospital	1. Short	9.72	3.11	11.057	0.000 ***	3 > 1
						3 > 2
						2 > 1
	2. Moderate	10.46	3.77			
	3. Long	12.89	6.03			

* *p* < 0.05, ** *p* < 0.01, *** *p* < 0.001.

**Table 3:** Summary of correlation for CIFS scores and social media use.

	1	2	3	4	5	6	7	8
1. CIFS Scores	1							
2. FB_usage	0.025	1						
3. IG_usage	0.003	0.526 **	1					
4. FB_Covid_post	0.117 **	0.206 **	0.137 **	1				
5. IG_Covid_post	0.169 **	0.035	0.212 **	0.392 **	1			
6. IM_Covid_post	0.140 **	0.146 **	0.152 **	0.344 **	0.325 **	1		
7. SM_Repost	0.497 **	0.064 *	−0.014	0.199 **	0.258 **	0.135 **	1	
8. IM_Repost	0.447 **	0.028	−0.021	0.159 **	0.178 **	0.180 **	0.566 **	1

FB_usage = Facebook usage time, IG_usage = Instagram usage time, FB_Covid_post = Number of COVID-19-related posts read daily on Facebook, IG_Covid_post = number of COVID-19-related posts read daily on IG, IM_Covid_post = number of COVID-19-related posts read daily on instant messaging, SM_Repost = frequency of reposting COVID-19-related information on social media, IM_Repost = frequency of reposting COVID-19-related information on instant messaging. * *p* < 0.05, ** *p* < 0.01.
